# Nephron Patterning: Lessons from *Xenopus*, Zebrafish, and Mouse Studies

**DOI:** 10.3390/cells4030483

**Published:** 2015-09-11

**Authors:** Audrey Desgrange, Silvia Cereghini

**Affiliations:** 1 Sorbonne Universités, UPMC Université Paris 06, IBPS–UMR7622, Paris F-75005, France; E-Mail: audrey.desgrange@upmc.fr; 2 Developmental Biology Laboratory CNRS, UMR7622, Institut de Biologie Paris-Seine (IBPS), Paris F-75005, France; 3 INSERM, U1156, Paris F-75005, France

**Keywords:** pronephros, metanephros, nephron segmentation, regulatory networks, vertebrates

## Abstract

The nephron is the basic structural and functional unit of the vertebrate kidney. To ensure kidney functions, the nephrons possess a highly segmental organization where each segment is specialized for the secretion and reabsorption of particular solutes. During embryogenesis, nephron progenitors undergo a mesenchymal-to-epithelial transition (MET) and acquire different segment-specific cell fates along the proximo-distal axis of the nephron. Even if the morphological changes occurring during nephrogenesis are characterized, the regulatory networks driving nephron segmentation are still poorly understood. Interestingly, several studies have shown that the pronephric nephrons in *Xenopus* and zebrafish are segmented in a similar fashion as the mouse metanephric nephrons. Here we review functional and molecular aspects of nephron segmentation with a particular interest on the signaling molecules and transcription factors recently implicated in kidney development in these three different vertebrate model organisms. A complete understanding of the mechanisms underlying nephrogenesis in different model organisms will provide novel insights on the etiology of several human renal diseases.

## 1. Introduction

All vertebrates possess an excretory organ, the kidney that regulates fluid balance, osmolarity and pH but also performs blood filtration in order to excrete metabolism end products and drugs. Most of these diverse tasks are accomplished by the nephrons, the functional units of the kidney.

During vertebrate development, the kidney emerges from intermediate mesenchyme (IM) and progresses either via two or three stages: pronephros, mesonephros, and metanephros. Although each of these kidney forms differs in their overall organization and complexity, they all have the nephron as their basic structural and functional unit.

Nephrons in amphibians, fish, and mammals are organized into discrete segments that are composed of distinct renal epithelial cell types carrying highly specific functions for glucose, solute transport and acid/base balance. Therefore, correct segmentation of the nephron is crucial for kidney function. The similarity in segmental organization of nephrons existing between the mammalian metanephric kidney and the frog and fish pronephros raises new possibilities to explore and dissect regulatory pathways controlling nephron patterning. This article reviews recent advances on nephron patterning in *Xenopus*, zebrafish, and mouse.

## 2. Kidney Morphogenesis in Non-Amniotes and Amniotes

In lower vertebrates, such as amphibians and fish, pronephric kidney is functional during embryonic and larval life and consists of two bilateral nephrons. Mesonephros will develop later on and additional nephrons will appear to ensure appropriate level of waste excretion in adult organisms. In amniotes, such as birds, reptiles, or mammals pronephros and mesonephros are only transient structures that will induce the formation of the functional metanephric kidney. Interestingly, pro-, meso-, and metanephros share similar organization: a filtration unit, a nephron tubule for solutes reabsorption and secretion, and a collecting duct to transport the filtrate product to the excretion site.

**Figure 1:**
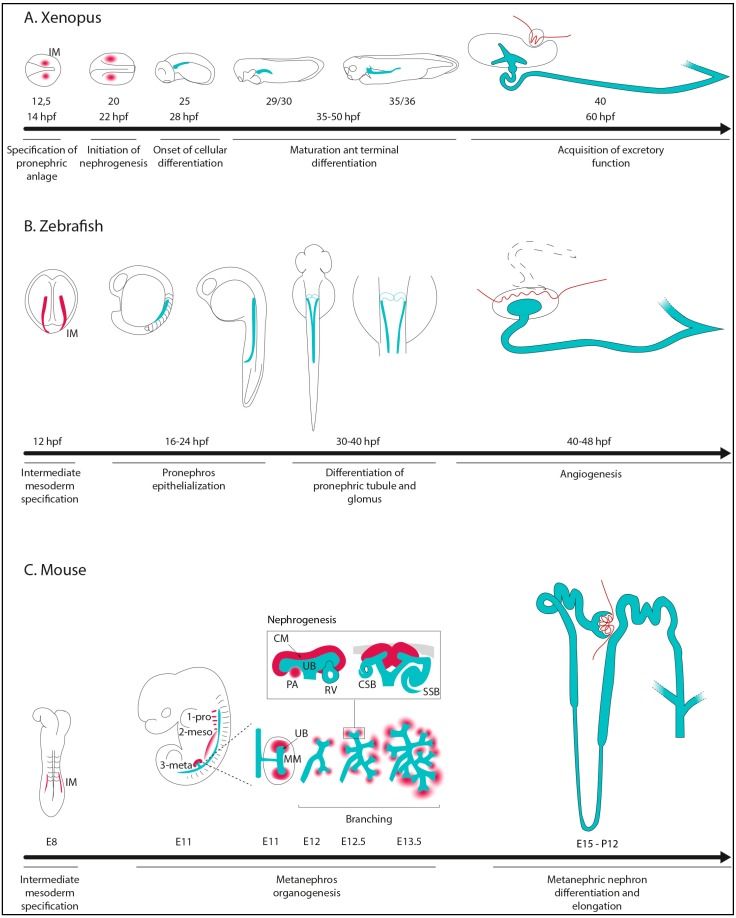
Stages of kidney morphogenesis in *Xenopus* (**A**); zebrafish (**B**), and mouse (**C**). hpf: hours post-fertilization; IM: Intermediate Mesoderm; pro-: pronephros; meso-: mesonephros; meta-: metanephros; UB: Ureteric Bud; MM: Metanephric Mesenchyme; CM: Cap Mesenchyme; PA: Pretubular Agregate; RV: Renal Vesicle; CSB: Comma-Shaped-Body; SSB: S-Shaped-Body. IM, CM, and PA are shown in red, while renal epithelial tubular structures are in light blue.

The pronephros in *Xenopus* is a simple nonintegrated nephron composed of three major compartments: the glomus, the tubule, and the duct. In contrast to zebrafish and mammals, the glomus does not connect with the most proximal part of the tubule but projects into the coelomic cavity, where the filtered blood is released. The tubules are formed opposed to the glomus. The pronephric anlagen are first induced from the intermediate mesoderm at about stage 12.5 (early neurula) by surrounding tissues to form a proximal condensate. This structure elongates into the dorso-ventral direction establishing the future pronephric tubule. By stage 24 (tailbud), the cells undergo a mesenchymal-to-epithelial transition (MET) to form an epithelial tubule, the pronephric duct. This duct elongates, becomes highly folded in the proximal region, while it grows caudally and fuses to the rectal primordium to subsequently open to the exterior via the cloaca. In the most proximal region of the pronephros, three ciliated funnels, the nephrostomes, open into the coelom and force coelomic waste into the proximal tubules. The glomus morphogenesis starts at stages 29–30 (late tailbud): a heap of capillaries arises from the dorsal aorta and associates with splanchnopleuric cells budding into the coelom. The blood starts to be filtered at stages 35–36 (tadpole) [1,2] (Figure 1A).

In contrast to *Xenopus*, the zebrafish pronephros comprises a pair of nephron tubules attached at their anterior ends to a single glomerulus. The pronephros arises from bilateral stripes of a renal progenitor field, emerging from the intermediate mesoderm precursors 12 hours post-fertilization (hpf). These cells are localized adjacent to a cell-field that generates a mixture of angioblasts and primitive blood cells. The pronephric tubule differentiates between 16 and 24 hpf through an antero-posterior MET transition process. The most proximal part of the nephron arises from the IM under somites 3 to 8, whereas the distal part comes from a small portion of tubules forming along the IM (Figure 1B). Thus, in zebrafish the IM contributes at all axial levels to the pronephros, while in other non-teleost vertebrates only the anterior part of the IM adopts a renal fate and the developing epithelial duct elongates to the cloaca. Moreover, epithelialization occurs simultaneously with patterning events and the establishment of distinct epithelial cell types. Further differentiation of the pronephric tubule occurs between 30 and 40 hpf. Imaging studies have also shown that fluid flow-induced collective tubule cell migration accounts for the convoluted shape of mature proximal tubules and the final position of the nephron boundaries [3].

Bilateral glomerular primordia fuse at 36–40 hpf ventral to the notochord, making the connection with the dorsal aorta and giving rise to an unique filtration unit. Between 40 and 48 hpf, capillaries bud from the aorta and the glomerular filtration begins around 48 hpf. Full maturation takes place until four days post-fertilization (dpf), together with appearance of well-developed podocytes [4,5] (Figure 1B).

The zebrafish mesonephros will form during the juvenile stages around the length of the pronephros, which acts as a scaffold for mesonephros formation [6]. It contains hundreds of nephrons and serves as the final adult kidney. Mesonephrogenesis continues throughout the life of zebrafish, with a rapid growth phase during the juvenile period and a slower phase in adulthood. This allows that the total nephron number of juvenile and adult fish correlates with the body mass [7].

In mice, the first morphological renal development hint occurs at E8 when paired epithelial nephric ducts (ND) (or Wolffian ducts) arise dorsally from the IM under the fifth somite. These bilateral ducts then reach the cloaca. As the ducts grow, they sequentially induce the formation of pronephric and mesonephric tubules from the adjacent IM. At the most anterior part, few mesenchymal cells adjacent to the nephric duct form rudimentary tubules, referred to as pronephros, which is a transient structure not functional in mammals. As pronephros degenerates, mesenchymal cells of the mid part of the ND (also often referred to as mesonephric duct) condensate and give rise to mesonephric tubules. These tubules, consisting of well-developed glomeruli and proximal tubule like structures, are transient filtration units that start to degenerate as the metanephric or definitive kidney develops [8,9].

Even if subsequently the mesonephric duct regresses, it has additional inductive functions for Mullerian duct development, while parts of its ductal system participate in the male reproductive organs. The Mullerian duct forms at embryonic day 11.5 (E11.5) close to the cranial part of the ND. Caudal elongation and maintenance of the Mullerian duct depends on ND integrity. In males, the Mullerian duct degenerates and the Wolffian duct (or ND) gives rise to the vas deferens, epididymis, and the seminal vesicles. In females, oviducts, uterine horns and the upper part of the vagina differentiate from the Mullerian duct while the Wollfian duct degenerates [10].

The definitive kidney, or metanephros, arises from the caudal part of the Wolffian duct under 25th somite by reciprocal interactions between the epithelium and the adjacent condensed mesenchyme. At E11, the ureteric bud emerges from the ND and invades metanephric mesenchyme (MM). Signals coming from both the ureteric bud and MM are necessary for the bud to extend and branch and subsequently give rise to the entire collecting duct system. Each time a new branch is formed, signals from the UB tip epithelium induce a subset of surrounding mesenchymal cells (cap mesenchyme) to undergo an epithelial transition in order to establish a polarized renal vesicle (RV) [11]. The distal part of the RV grows and connects to the adjacent UB epithelium and rapidly evolves to form the Comma- and then the S-shaped bodies (CSB and SSB). The SSB is a highly polarized structure composed of the proximal, intermediate, and distal segments, the latter connects to the UB epithelium. The most proximal segment is further subdivided into two epithelial layers, the parietal (Bowman capsule) and visceral (podocyte). The SSB grows and further differentiates to form a mature nephron composed successively by the glomerulus, the proximal tubule, the loop of Henle, and the distal tubule. Mature nephrons are observed by E16.5 in mice, but mesenchyme aggregation continues until postnatal day two (P2). Thus, several nephrogenesis stages coexist at the same developmental stage [12]. In mice, approximately 12,000 nephrons will arise by this process in each metanephros, whereas in humans the average number of nephrons per kidney is close to one million [9]. The ureter will develop from the most proximal part of the UB and then connect to the bladder at E13 [13] (Figure 1C). By postnatal day three (P3) the pool of nephron progenitors is exhausted and both branching and nephrogenesis stop.

Therefore, even if the non-amniotes pronephros and the amniotes metanephros have different shapes and adaptive functions, their development is similar and occurs through four stages:

1. Specification of IM cells and renal primordium formation.
2. Epithelialization and differentiation of renal primordium.
3. Patterning of nephrons to make functional and specialized segment tubules.
4. Formation of glomerular capillaries for blood filtration.

In the case of the pronephric kidney, these steps will occur only once to form one pair of bilateral nephrons. In mammals, these processes will repeat numerous times to give rise to a highly branched system connected to thousands of nephrons.

## 3. Conservation of the Segmental Organization of Nephrons in Vertebrates

**Figure 2:**
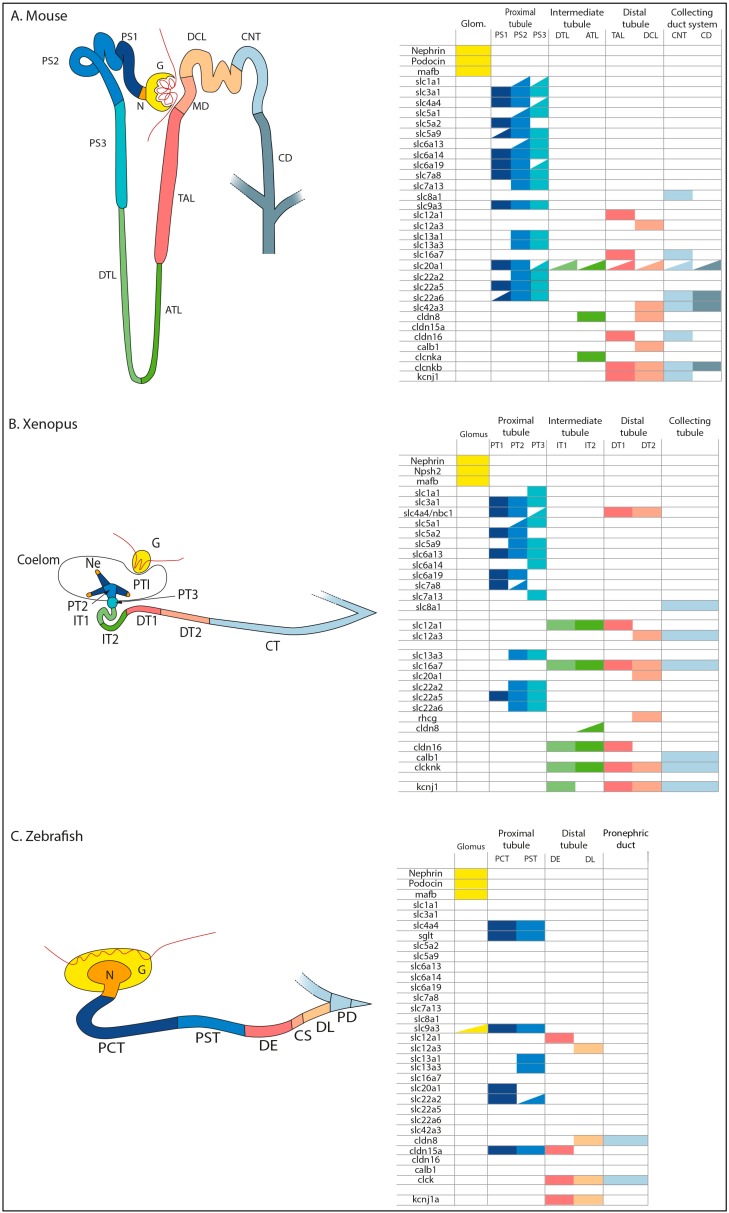
Nephron segments and table of expression of marker genes. The different segments are depicted schematically with different colors showing the co-expression of selected markers in different segments in mature mouse/mammalian metanephric kidney (**A**), *Xenopus* pronephros (**B**), and zebrafish pronephros (**C**). Mature nephrons are observed from E16.5 in mouse, from 60 hpf in *Xenopus* pronephros and from 40 hpf in zebrafish. Segment names are abbreviated as follows: in mouse, G: glomerulus; N: neck; PS1, PS2, and PS3: segments of the proximal tubule, DTL: descending thin limb, ATL: ascending thin limb; TAL, thick ascending limb, MD: macula densa, DCT: distal convoluted tubule, CNT: connecting tubule; CD: collecting duct; in *Xenopus*, G: glomus; Ne: nephrostomes; PT1, PT2, and PT3: segments of proximal tubule; IT1 and IT2: segments of intermediate tubule; DT1 and DT2: segments of distal tubule; CT: collecting tubule; in zebrafish, G: glomerulus; N: neck; PCT: proximal convoluted tubule; PST: proximal straight tubule; DE: distal early; CS: Corpuscule of Stannius; DL: distal late; PD: pronephric duct. Yellow: glomerulus (in mouse and zebrafish) or glomus (in *Xenopus*); orange: neck (in mouse and zebrafish) or nephrostomes (in *Xenopus*); blue: proximal segments; green: intermediate segments; pink: distal segments; gray: duct. Half colored boxes indicate low expression.

In mammals, proximal, intermediate, and distal tubules are three major nephron segments but they can be further subdivided in regard to their morphology and molecular signature. The glomerulus is connected to the proximal convoluted tubule by the neck. The proximal tubule can be subdivided into three segments: the convoluted tubule is formed by segments S1 (PS1) and S2 (PS2) and is followed by the straight tubule (or S3). The loop of Henle is further divided into the descending thin limb (DTL) and the ascending thin limb (ATL). The distal tubule comprises the thick ascending limb (TAL) and the distal convoluted tubule (DCT). The connecting tubule makes the connection between the nephron and the collecting duct. Terminal differentiated metanephric nephrons are therefore highly specialized structures characterized by a specific combination of solute transporters and tight junction elements. Several studies have pointed to the regional expression of solute transporters along the proximo-distal axis of metanephric kidney. In particular, the solute carrier (*Slc*) gene family appears to be expressed in similar fashion among vertebrates [14,15,16,17] (Figure 2A). Vertebrate nephrons are also characterized by the regional expression of tight junction components, such as Claudins and Occludins. Tight junctions enable the relatively leaky proximal tubule segments to reabsorb solutes and in the distal tubule segments to tightly regulate solute movement for fine-tuning salt and electrolyte levels in the body [18].

In amphibians, the segmental organization of the pronephros has been first appreciated thanks to its histological and physiological features [19]. As in mammals, the proximal tubule reabsorbs sodium, amino acids, and glucose. The pronephric intermediate part seems to be specialized in the reabsorption of minerals and ions. In contrast to the mammalian collecting duct, the pronephric duct is not specialized for urine concentration. This comes from the fact that *Xenopus* and other aquatic amphibians do not excrete urea but ammonia [20,21].

Expression studies, mostly based on *slc* gene expression mapping as well as other transporters, have shown that the *Xenopus* pronephric tubule can be divided into at least four distinct domains [21,22,23,24,25]. More recently, a large-scale analysis has shed more light onto an even more complex structure with eight functionally distinct domains that share significant analogies with those observed in mammalian metanephric nephron [14]. In *Xenopus*, the proximal tubule (PT) is divided into three segments (PT1, PT2, and PT3), whereas the intermediate tubule (IT) and the distal tubule (DT) are both composed of two segments IT1 and IT2, and DT1 and DT2, respectively. In contrast, the connecting tubule does not appear to be further subdivided. As illustrates the schematic representation of nephron segments in Figure 2, the proximal tubule is the region that shares the highest degree of structural and functional similarity with the mammalian proximal tubule (Figure 2A,B). A very good example of solute carrier conservation is the low-affinity and high-affinity Na-glucose transporters *slc5a2* and *slc5a1* sequentially expressed along the proximo-distal axis of the proximal tubule [26]. Thus, *slc5a2* localizes to the S1 and S2 segments of the mouse proximal convoluted tubule and to the *Xenopus* PT1 and PT2 segments, whereas *slc5a1* is expressed in the mouse S3 and the *Xenopus* PT2 and PT3 segments. No water transporter was found in the *Xenopus* intermediate segment. Thus, urine is not concentrated within the intermediate tubule and stays hypo-osmotic with plasma, suggesting the absence of an intermediate tubule. However, the intermediate segment expresses *cldn8*, also present in the mouse descending thin limb. Moreover, functional studies have further shown that the Irx transcription factors are present in both organisms in the future intermediate segment (see below). The *Xenopus* intermediate pronephros displays in addition distal tubule characteristics, since it expresses markers such as *slc12a1*, *slc12a6*, or *kcnj1* [14]. The distal segment shares similarities with the mammalian TAL and DCT. Thus, the *Xenopus* DT1 and the mouse TAL segments express similar markers, such as the Na-K-Cl transporter *slc12a1*, while *slc12a3* (NCC) is restricted to the *Xenopus* DT2 and the mammalian DCT. Finally, as in the mouse, the *Xenopus* connecting tubule showed expression of the transporter *slc16a7* and the tight junction markers *calb1*, *clcnk*, and *kcnj1*. This further emphasizes the similarities between the connecting tubules of the pronephros and metanephros (Figure 2B). Up to now, there is no molecular evidence (transporters or cell-cell junctions) that may link *Xenopus* collecting tubule function to the mammalian collecting duct [14,15].

In zebrafish, interesting similarities have also been found in several differentiated cell types between pronephros and the mammalian metanephros. Within the glomeruli, endothelial cells, are fenestrated and podocytes interdigitate with foot processes [4]. At the molecular level, podocytes express hallmark components of the slit diaphragm, including the proteins nephrin and podocin. As in *Xenopus*, the zebrafish pronephros can be subdivided into at least eight discrete regions. The proximal convoluted tubule (PCT) displays an apical brush border and expresses transporters necessary for acid-base homeostasis as well as for glucose and amino acids reabsorption, such as in mammals’ PCT. The PCT and the proximal straight tubule (PST) express *slc9a3*, which is a proximal tubule marker in mammals [27].

Gene expression analyses have also demonstrated that two tight junction genes, *tjp2a* and *tjp3*, are expressed in the distal pronephros [28,29]. The distal tubule is composed by the distal early (DE) and distal late (DL) tubules. Interestingly, the DE expresses *slc12a1*, a marker of the mouse TAL, and the DL expresses markers that are also specifically found in mammalian DCT and collecting duct such as *clck*, *slc12a3*, and *cldn8* [27]. The thin limb segment is absent in zebrafish. As fish live in freshwater, they neither need to conserve water, nor to concentrate their urine. This further explains the shortness of the collecting tubule, as in *Xenopus* (Figure 2C).

Some transporters, however, display different expression domains between frog, zebrafish, and mammals. For instance, *slc20a1* is expressed in the distal segment in frogs, whereas its expression is restricted to the PT in zebrafish and mammals. Similarly, *slc4a4* is found in the PT of the three species, but in *Xenopus* is additionally expressed in the DT [22] (Figure 2B). Note that further studies are required to complete the expression pattern of several other nephron markers in the zebrafish pronephros (Figure 2C). Despite this, the studies described above clearly show that nephron segment organization and function is remarkably conserved among vertebrates. Thus, *Xenopus* and zebrafish are emerging as interesting models to dissect signaling pathways and transcriptional circuits controlling the nephron patterning in vertebrates.

## 4. Nephron Patterning/Segmentation: Genetic and Transcriptional Regulation

While there has been much progress in the understanding of the early steps of renal epithelial cell differentiation, a major gap remains in the comprehension of the regulatory cascades regulating nephron patterning. Interestingly, analogies in nephron segmentation between pro- and metanephros suggest that the cellular and molecular processes controlling nephron segmentation are conserved among the vertebrates. Indeed, many signaling molecules that govern pronephros formation have turned out to be evolutionarily conserved and have similar functions in the metanephric kidney.

In *Xenopus*, loss and gain of function studies have pointed to several transcription factors involved in transcriptional regulation of pronephros segmentation. It has been shown that the zinc finger transcription factor *evi1*, expressed in the distal tubule and duct, is required for patterning of these segments. Accordingly, *evi1* overexpression blocks proximal segment fates [23]. The Iroquois transcription factors *irx1*, *2*, and *3* are expressed in the proximal tubular segment PT3 and the intermediate tubule segments IT1 and IT2 [25]. Loss of function of *irx1* and *3* leads to a reduction of *slc7a13* and *slc12a1* expression, showing that both genes are required for intermediate tubule segment formation [25,30]. Notch signaling has also been shown to play a predominant role in patterning the *Xenopus* pronephros anlagen by regulating proximal fate. Indeed, *delta1*, *serrate1*, *notch1*, the glycosyl transferases lunatic fringe and radical fringe (*lfng*, *rfng*) determine whether cells are fated towards the proximal tubule or the distal tubule [31,32,33,34]. Several transcription factors genes are expressed in the developing podocytes, such as *wt1*, *foxc2*, *lmx1b*, *mafb*, *tcf21*, and *hey1*. Simultaneously, knockdown of *wt1* and *foxc2* leads to an absence of podocytes. The regulatory network appears to be highly orchestrated since only double or triple knockdowns of these transcription factors affect podocyte development [34]. The signals upstream these factors have only started to be investigated. It appears that *irx1* and *3* are regulated by retinoic acid (RA) during nephron segmentation [31]. However, RA function in *Xenopus* pronephros patterning remains unclear, since downregulation of RA leads to a failure of pronephros formation [35].

**Figure 3:**
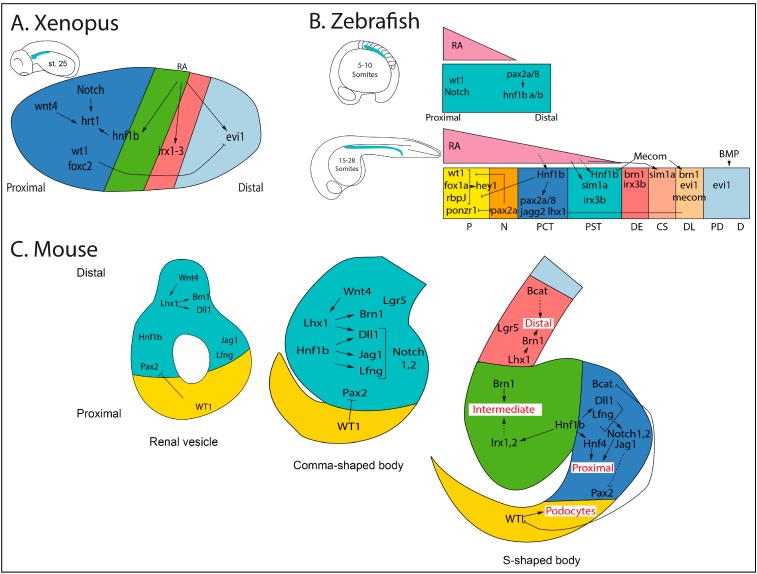
Genetic pathways of nephron segmentation in *Xenopus* (**A**), zebrafish, (**B**) and mouse (**C**). Colors are the same as described in Figure 2.

The transcription factor *hnf1b* is expressed in the pronephric field and is maintained throughout the entire pronephros with highest levels in the proximal part. We have recently shown that *hnf1b* is required for both proximal and intermediate tubule fates in *Xenopus*. Our own experiments further suggest that it may act on these patterning events through the Notch pathway and the Iroquois genes within a complex regulatory circuit [36]. The epistatic relationships between RA and *hnf1b* during nephron segmentation have not yet been determined. However, studies of the mouse *Hnf1b* promoter and enhancer have shown that *Hnf1b* is a direct target of RA [37,38]. Moreover, *hnf1b* acts downstream of RA in the developing *Xenopus* neural tube, further suggesting a similar regulatory circuit during pronephros segmentation [39] (Figure 3A).

During zebrafish pronephros development, IM initially expresses *pax2a*, *pax8*, and *lhx1* [40,41]. It then becomes rapidly subdivided into proximal and distal territories. The most anterior part of the IM starts to express *wt1a* and the Notch signaling components *deltaC*, *jagged1b*, *jagged2a*, *rbpJ*, and *hey1*. The rest of the tubule now displays different domains: *pax2a/8* and *jagged2b* are expressed in the proximal segment, while *irx3b*, *evi1*, and *pou3f3a*/*pou3f3b* are restricted to the distal territories [15,42] (Figure 3B). Thus, numerous ortholog genes are expressed in restricted segment domains suggesting that they may have a conserved role in nephron patterning.

It has been shown that *wt1a* is required for podocyte fate acquisition interacting with *foxc1* and *rbpj* to control the expression of podocyte marker genes and modulating Notch signaling [43,44]. There are also evidences that *pax2a* directly inhibits podocyte formation by antagonizing *wt1* activity and establishing a boundary between the podocyte and the neck. Reciprocally, the gene *ponzr1* modulates negatively *pax2a* to restrict its activity within the podocyte territory [45,46]. As in *Xenopus*, *irx3b* plays an essential role for DE segment patterning modulating the boundary between proximal and distal segments [47]. Interestingly, zebrafish embryos express *hnf1ba* and *hnf1bb* in the pronephric tubule. Its expression in the IM requires *pax2a* and *pax8*. Deficient embryos for *hnf1ba* and *b* fail to express proximal and distal segment markers and ectopically express podocyte markers. This shows that hnf1b transcription factors are required for nephron segmentation. They act in parallel with *wt1* and *rbpJ* to restrict podocyte formation and to control segment patterning through the regulation of *pax2a/8*, *jagged2a*, and *irx3b* [48,49].

Interestingly, RA deficient embryos fail to express *wt1a*, *deltaC*, and *jagged2a* in the proximal IM, whereas and *pou3f3a* and *pou3f3b* expression are expanded leading to a reduced number of podocytes, poorly developed proximal segments and extension of the distal segment [15,27,47]. Furthermore, it has been shown that *hnf1b* acts downstream RA to promote tubule differentiation [48]. These results suggest that RA acts as an organizing center, in a graded fashion, promoting proximal segment formation and limiting distal segment development. It has also been shown that the transcription factor *sim1a*, dynamically expressed in the renal field from caudal to proximal segments, is necessary to induce PST and the corpuscule of Stannius fates. *Sim1a* seems to act downstream of RA on the PCT/PST boundary establishment through the inhibition of the PCT fate [50]. The transcription factor Mecom, displaying a similar dynamic expression pattern to *sim1a*, has been shown to be required for distal tubule formation and restriction of proximal fates through the modulation of Notch signaling [27,47]. Thus, RA and *mecom* appear to play opposite roles in segment fate acquisition [51] (Figure 3B).

In mammals, the nephron induction process is mediated by interaction between MM and UB cells. Classical work of Grobstein and Saxén has led to the identification of canonical Wnt signaling coming from the UB as the primary inductive event of nephrogenesis [8,52,53]. *Wnt9b* activates *Wnt4* expression within the mesenchymal pretubular aggregates, which further epithelialize them [54,55]. In parallel, *Wnt9b* also insures the expression of *Six2* in the cap mesenchyme to maintain a self-renewing nephron progenitor pool [56].

The first indication of polarity and proximo-distal axis appears quite early. Indeed, just after MET, several genes begin to be expressed in a polarized manner in the RV. The distal domain is defined by the restricted expression of many genes, including the transcription factors *Lhx1* and *Brn1 (Pou3f3)*, the Notch ligands *Dll1* and *Jag1*, as well as *Bmp2*, whereas the proximal domain is characterized by the high expression of *Wt1* [57,58,59]. Consistent with these expression patterns, *Wt1* is required for glomerulus podocyte layer specification [60]. The transcription factor *Pax2* exhibits segmented expression during nephron morphogenesis and is strongly reduced in the podocyte progenitor territory, where *Wt1* expression is high. It has been shown that *Wt1* promotes podocyte development through direct repression of proximal *Pax2* activity [61]. *Lhx1*-deficient RVs fail to regionalize along the proximo-distal axis, lack the expression of the transcriptional targets *Brn1* and *Dll1*, and do not progress to the CSB stage [58]. *Brn1*, in turn, is involved in loop of Henle elongation and distal convoluted tubule formation [57]. These observations show that *Brn1* and *Lhx1* are required for distal and medial nephron segment formation. It is still unclear what are the first signals driving this polarization. However, polarized expression of *Wnt4* and *Lef1* within renal vesicle suggests that a *Wnt*-signaling gradient may contribute to this process [62] (Figure 3C). Future nephron segmentation becomes more evident at the SSB stage, where discrete domains are recognizable by the regionalized expression of several key markers.

Several studies have implicated the Notch pathway in podocyte and proximal tubule fate acquisition. Accordingly, Notch pathway components display polarized expression patterns within the SSB: the Notch ligands *Dll1*, *Jag1* and the modulator of the pathway *Lfng* are expressed in the proximo-median region [58,63] (Figure 3C). Interestingly, either loss of Notch signaling, disruption of *Notch2* or *RbpJ* expression or inhibition of gamma-secretase or presenilins, led to abnormal nephrons lacking podocytes and proximal tubules. On the other hand, ectopic activation of the Notch pathway promotes the formation of proximal tubule cells [64,65,66,67]. Consistent with the role of the Notch pathway in proximal fate acquisition, *Dll1* hypomorphs show a reduction of proximal tubule [66]. Moreover, conditional invalidation of *Dll1* in pretubular aggregates is associated with a reduced length of the proximal tubule with no effect on podocyte development (A.D. and SC unpublished results). Recent studies have further shown that the ligand *Jag1* plays a dominant role relative to Dll1 for both podocyte and proximal tubule development [68].

Interestingly, members of the Iroquois gene family *Irx1*, *Irx2*, and *Irx3* are expressed in a highly restricted manner in the intermediate segment of the mammalian SSB, suggesting a similar function as in *Xenopus* or zebrafish [24]. We have recently shown that the transcription factor *Hnf1b* is required for correct patterning of early nephron structures. Inactivation of *Hnf1b* in pretubular aggregates leads to defective morphology of SSBs, associated with the downregulation of the Notch components *Dll1*, *Lfng*, *Jag1* as well as the transcription factors *Irx1* and *Irx2* (Figure 3C). Moreover, *Hnf1b* is recruited to the regulatory sequences of most of these genes [35]. Interestingly, *Hnf1b* inactivation in the early nephron progenitors (cap mesenchyme) leads to similar defects in nephron segmentation [69]. These results show that *Hnf1b* is required for the acquisition of a proximo-intermediate segment fate in the vertebrates, thus uncovering a previously unappreciated function of a novel SSB subcompartment in global nephron segmentation and further differentiation.

The surface receptor *Lgr5* is expressed in the distal segment of the SSB and in the distal convoluted tubule and thick ascending limb of the nephron. Lineage tracing studies have shown that *Lgr5* marks progenitor cells of these segments. *Lgr5* deletion did not lead to any kidney phenotype, but *Lgr4/5* double knockout causes the loss of distal progenitors and reduced proliferation in the nephron tubules [70,71]. A large-scale *in situ* hybridization analysis has shown that *Evi1* is expressed in the developing SSB [16]. Unfortunately the early lethality of *Evi1*-null embryos impairs further analysis of its role in nephron patterning [72].

More recently, a β-catenin signaling gradient has been involved into nephron patterning. Pharmacological inhibition of β-catenin activity favored proximal cell differentiation. Reciprocally, proximal fate was repressed when β-catenin activity was increased [73] (Figure 3C). Consistent with these observations, studies on the chick mesonephros have recently shown that *Wnt*-signaling patterns the proximo-distal axis of the nephron, with proximal regions differentiated in regions with lowest *Wnt* signaling [74]. However, it remains unclear which are the regulators establishing the *Wnt*-β-catenin gradient and which are the direct targets involved in nephron patterning.

In contrast to *Xenopus* and zebrafish nephron patterning, RA has not been linked to proximo-distal patterning of the mouse metanephric nephron. Further studies are needed to elucidate the possible involvement of RA signaling in nephron segmentation, since *Raldh1* and *2* are expressed in a restricted manner in the rat nephric duct and early nephron structures [75].

## 5. Conclusions and Future Prospects

Nephron patterning is a complex process that ensures the formation of highly specialized segments and their following physiological functions. The fundamental conservation of molecular anatomy between pronephric and metanephric nephrons has a great potential for discovering genes and regulatory pathways during nephron segments establishment. *Xenopus* and zebrafish studies are therefore crucial for our understanding of this critical field in developmental biology. These studies show that the molecular circuits involved in the proximo-distal nephron patterning in vertebrates are highly similar. Nevertheless they are not completely conserved, in particular the timing of appearance and/or requirement of some regulators. Several invertebrate models have also been studied, such as the fruit fly *D. melanogaster*, and obtained results that are further highlighting the amazing degree of conservation among the excretory cell types across the phylogenetic tree [76].

Although the key transcriptional players of nephron segmentation have been identified, many of them remain to be discovered as well as the epistatic relationships and interactions that may exist among them. An additional and important issue will be the identification of the upstream signaling pathways acting on key regulators to establish distinct nephron segment fates. Systematic approaches such as RNA-seq and ChIP-seq on specific nephron segment progenitors combined with the use of simpler vertebrate models will certainly provide new insights onto the regulatory programs underlying theprocess of nephron segmentation.
